# The Impact of the Soil Survival of the Pathogen of *Fusarium* Wilt on Soil Nutrient Cycling Mediated by Microorganisms

**DOI:** 10.3390/microorganisms11092207

**Published:** 2023-08-31

**Authors:** Xuecheng Yan, Shuhan Guo, Kexiang Gao, Shuaibin Sun, Chenglin Yin, Yehan Tian

**Affiliations:** Shandong Provincial Key Laboratory for Biology of Vegetable Diseases and Insect Pests, College of Plant Protection, Shandong Agricultural University, Taian 271018, China; yanxuecheng97@163.com (X.Y.); gsh13954949468ao@163.com (S.G.); kxgao63@163.com (K.G.); shuaibinsun@163.com (S.S.); yin1990610@163.com (C.Y.)

**Keywords:** *Momordica charantia*, *Fusarium* wilt, *Fusarium oxysporum*, spatial distribution, soil nutrient cycling

## Abstract

*Fusarium* wilt of *Momordica charantia* in the greenhouse is one of the most severe crop diseases in Shandong Province, P.R. China. This study aimed to investigate the mechanisms of accumulation and long-term survival of the pathogen in naturally pathogenic soils. Soil physicochemical properties were tested after applying a highly virulent strain of *Fusarium* wilt to *M. charantia* in an artificial disease nursery. The functional structure of soil microorganisms was analyzed through amplicon sequencing. The highly virulent strain SG−15 of *F. oxysporum* f. sp. momordicae was found to cause *Fusarium* wilt in *M. charantia* in Shandong Province. The strain SG−15 could not infect 14 non-host crops, including *Solanum melongena* and *Lycopersicon esculentum,* but it had varying degrees of pathogenicity towards 11 *M. charantia* varieties. In the artificial disease nursery for *Fusarium* wilt of *M. charantia*, the *F. oxysporum* was distributed in the soil to a depth of 0–40 cm and was mainly distributed in crop residues at 0–10 cm depth. During crop growth, *F. oxysporum* primarily grows and reproduces in susceptible host plants, rather than disease-resistant hosts and non-host crops. The colonization of the pathogen of *Fusarium* wilt significantly changed the soil physicochemical properties, the functional structure of soil microorganisms and the circulation of soil elements such as carbon, nitrogen, phosphorus and sulfur. Soil pH value, organic matter content, available iron content, available manganese content, FDA hydrolase activity and polyphenol oxidase activity were significantly correlated with the relative abundance of *Fusarium* wilt pathogens in the soil. In general, this study suggests that susceptible host plants facilitate the accumulation of *Fusarium* wilt pathogens in the soil. These pathogens can mediate the decomposition process of plant residues, particularly those of diseased plants, and indirectly or directly affect soil’s chemical properties.

## 1. Introduction

*Fusarium wilt* is a soil-borne disease of crops caused by the pathogenic *Fusarium oxysporum*. It has become one of the most harmful plant diseases in the world and increasing yearly. The pathogenic *F. oxysporum* can harm over 100 essential cash crops such as banana, tomato, cucumber, *M. charantia*, watermelon, muskmelon, cotton and beans. It has gradually expanded from the affected to the non-affected areas worldwide, becoming a crucial obstacle to developing the world’s cash crop industry [1,2,3,4]. However, the research on the destructive mechanism of crop *Fusarium* wilt needs to be more comprehensive, and the prevention and control measures need to be more systematic. Hence, there is an urgent need to investigate the incidence of *Fusarium* wilt, identify the weak points in the disease cycle of *Fusarium* wilt and develop efficient ways to detect and curb the spread of *Fusarium* wilt quickly.

*Fusarium oxysporum* is a typical soil-dwelling fungus that can survive winter in soil or on diseased plant debris and has a strong saprophytic effect on organic matter. In the absence of the susceptible host plant, it can live saprophytically and has intense competition with other saprophytic fungi in soil [5,6]. *F. oxysporum* is predominant in dying host plants and can grow unhindered in the roots of the infected plant; when the host plant dies, the roots infected with the fungus will decay and release spores into the soil [6,7]. In the decay stage of residual underground muskmelon roots, the dominant position of *F. oxysporum* in the plant residues is not easily replaced by other saprophytic fungi in the soil, and it can survive in the soil for several years under the protection of plant residues [8,9]. The persistence of pathogenic *F. oxysporum* in the soil is affected by the host plant residues and its decay rate. The population of pathogenic *F. oxysporum* decreases naturally as its propagation vectors are consumed, but the pathogen may persist at low levels for several years [10,11]. The lack of protection from host plant residues may decrease the number of pathogens in the soil [12].

The occurrence of crop *Fusarium* wilt is determined by the number of pathogens in the soil and the relationship with soil physicochemical properties, such as pH, organic matter and nutrient content [13]. Metagenomic sequencing showed that the community composition of *Fusarium* spp. varied between species in a monoculture plant model. *F. oxysporum* was the dominant fungus, and its presence was positively or negatively correlated with soil physical and chemical properties. Studies have shown that soil available iron content (AFe) is higher in areas with a high incidence of banana *Fusarium* wilt. The pH value and available phosphorus (AP) content in healthy soils were significantly higher than those in pathogenic soil [14]. In contrast, the total organic carbon (TOC), total organic nitrogen (TON) and available potassium (AK) content in healthy soil were significantly lower than those in pathogenic soil [15]. The occurrence of crop *Fusarium* wilt is closely related to the amounts and invasion of soil pathogens and the physicochemical properties of soil. Exploring the nutrient characteristics of pathogenic soil and their relationship with pathogens is crucial for preventing and controlling soil-borne diseases.

The study aims to compare soil physicochemical properties and spatial distribution of *F. oxysporum* with and without *Fusarium* wilt in the same field, analyze soil property changes after pathogen invasion, and reveal the relationship between soil nutrient characteristics and pathogens through *Fusarium* wilt pathogen inoculation and preparation of an artificial disease nursery. The results will provide a more scientific and rational approach toward the prevention and management of *Fusarium* wilt and the suppression of soil-borne diseases.

## 2. Materials and Methods

The experiment was conducted from September 2014 to December 2021 in Shouguang and Feicheng, Shandong Province and the Plant Protection Experimental Station at Shandong Agricultural University.

### 2.1. Plants and Pathogen

*M. charantia* hybrids for the test including Ruyu−41; Ruyu−1420; Ruyu−1510; Ruyu−1511; Ruyu−1512; Ruyu−1515; Ruyu−1516; Ruyu−1522; Ruyu−1523; Ruyu−1534; and Ruyu−1536 were gifted from Prof. Yucan Zhang of Fujian Academy of Agricultural Sciences. A pathogen assay was conducted using *Fusarium oxysporum* f. sp. *momordicae* strain SG−15 (ACCC 39204). The pathogen was isolated and purified from diseased *M. charantia* collected from Shouguang, which is the main production area of *M. charantia* in protected vegetable cultivation in Shandong Province. The isolated pathogen was stored in our laboratory.

### 2.2. Fungus Isolation and Pathogenicity Test

The fresh *M. charantias* with *Fusarium* wilt symptoms were collected from the *M. charantia* greenhouse in Shandong Province. We collected 24 diseased *M. charantia* tissues from 8 greenhouses for pathogen isolation and identification in Shouguang City (6 greenhouses) and Feicheng City (2 greenhouses). The diseased plant tissues were washed with tap water and dried. The brown stain vascular bundles inside *M. charantia* were screened by the plant tissue separation method and cultured in a PDA medium for 3–5 days. Next, 15 tissues at the disease–health junction were collected for each plant and evenly placed in 3 plates. The suspected pathogenic strains of *Fusarium* wilt of *M. charantia* were obtained by single spore isolation and purification method. The pathogenicity testing was performed according to Koch’s postulates. The suspected pathogenic strains were inoculated into 33 healthy *M. charantias* seedlings with 2–4 leaves. After the occurrence of *Fusarium* wilt in the inoculated *M. charantia* seedlings were obtained by tissue isolation, purification, single spore isolation, and cultivation. Finally, we identified the suspected pathogenic strains of *Fusarium* wilt of *M. charantia* by comparing the colony morphology with the original strain.

### 2.3. Establishing the Artificial Disease Nursery for Fusarium wilt of M. charantia

In order to better investigate the causes of *Fusarium* wilt outbreak in *M. charantias* and the spatial distribution of the *Fusarium* wilt pathogen, in 2015 the experimental group of this research established an artificial disease nursery for *Fusarium* wilt of *M. charantia* at the Plant Protection Experimental Station of Shandong Agricultural University (36°18′ N, 117°17′ E). The region has a warm temperate monsoon climate, with an annual average sunshine number of 2627.1 h. The average annual temperature is 12.9 °C, and the average annual precipitation over the years is 697 mm. Preparation of plot model with a high incidence of *Fusarium* wilt of *M. charantia*: ① The highly virulent SG−15 strain of *Fusarium* wilt was propagated using potato dextrose broth medium to create a pathogen spore suspension for *M. charantia*. The spore suspension was diluted 100 times with tap water and mixed with peat soil (1:1 W/V). The second propagation was incubated for 30 days. The soil was then evenly scattered with plant residues containing pathogens (about 1.0 × 10^4^ spores·g^−1^), and the disease nursery (1 kg·m^−2^) was prepared by rotary tillage before incubating for 30 days. ② *M. charantia* was cultivated twice a year for five years. A plate dilution and coating method was used to count the number of pathogens of *Fusarium* wilt in plant residues. Briefly, 1 g of plant residue samples were added to 99 mL of sterile water and mixed thoroughly. About 1 mL of this suspension was added into 9 mL of sterile water to make 10^−3^ suspension, and then 0.2 mL of 10^−3^ suspension was spread on the Komada medium [16]. The average number of pathogens of *Fusarium* wilt in plant residues was obtained after repeating the above steps 5–6 times.

### 2.4. Field Pathogenicity Assay of SG−15 Strain

From June 2018 to October 2020, seedlings with 2–4 leaves stage of *Momordica charantia*, *Benincasa hispida*, *Cucurbita pepo*, *Luffa cylindrica*, *Cucumis melo*, *Cucumis sativus*, *Lagenaria siceraria*, *Solanum melongena*, *Lycopersicon esculentum*, *Capsicum annuum*, *Abelmoschus esculentus*, *Lablab purpureus*, *Brassica pekinensis*, *Raphanus sativus*, *Zingiber officinale* and Ipomoea batatas were selected and planted in the artificial disease nursery for *Fusarium* wilt of *M. charantia*. Each crop was planted in three independent experimental plots and each experimental plot had an area of 20 m^2^ with 16 crop species. The incidence of *Fusarium* wilt in the field was investigated at 30 days and 45 days after transplanting. Disease severity of *Fusarium* wilt of *M. charantia* was scored according to the classification standard of Chen [17]. The experiment was repeated for three years.

### 2.5. Distribution of Fusarium oxysporum in Soil

Metagenomic and dilution plate coating methods were used to count the quantity of *F*. *oxysporum* in the artificial disease nursery soil of *Fusarium* wilt of *M. charantia*. (1) The distribution of *F*. *oxysporum* in different depth soil layers: five sampling points were set on the artificial disease nursery for *Fusarium* wilt. Soils samples were collected from five soil layers (i.e., 4–6, 9–11, 19–21, 29–31, and 40–45 cm) at each of the 5 plots using a five-point method denoted as D5, D10, D20, D30 and D40, respectively. The soil layer was sampled five times and combined into one mixed soil sample. Each soil treatment had five independent experimental replicates. After drying at room temperature, the number of *F. oxysporum* was counted using dilution plate coating and metagenomic methods [18]. (2) The distribution of *F*. *oxysporum* in the rhizosphere soil and roots of different resistant hosts: the susceptible *M. charantia* (Ruyu−41) and disease-resistant *M. charantia* (Ruyu−1512) were planted in the artificial disease nursery for *Fusarium* wilt of *M. charantia*. After 45 days of planting, the root tissues and the rhizosphere soil from 25 plants, each of Ruyu−41 and Ruyu−1512, were randomly collected to analyze the number of *Fusarium*, denoted as S-root (root tissue of susceptible *M. charantia*), R-root (root tissue of disease-resistant *M. charantia*), S-soil (the rhizosphere soil of susceptible *M. charantia*) and R-soil (the rhizosphere soil of disease-resistant *M. charantia*), respectively. Moreover, each treatment contains 5 independent replicates of 5 plant root tissues or soil. After drying the root tissue and rhizosphere soil samples at room temperature, the number of *F. oxysporum* was counted using the dilution plate coating method. (3) The distribution of *F*. *oxysporum* in plant residues: after 3 months of crop harvest, the root tissue residues and rhizosphere soil from 25 plants of susceptible *M. charantias* (host) and non-host plants such as eggplant (non-host) were randomly collected to analyze the number of *F*. *oxysporum*, denoted as host (the root tissue residues of *M. charantias*), non-host (the root tissue residues of eggplant), P-soil (the rhizosphere soil of *M. charantia* and eggplant in the artificial disease nursery for *Fusarium* wilt) and H-soil (CK, the rhizosphere soil of *M. charantia* in healthy soil). Each treatment had 5 replicates with 5 plant root tissues or soil samples. After drying at room temperature, *F*. *oxysporum* was counted using the dilution plate coating method. 

### 2.6. Effects of the Colonization of F. oxysporum on Soil Physicochemical Properties and Microbial Function

Five sampling points were set at the artificial disease nursery for *Fusarium* wilt-treated and the *Fusarium*-free regions, respectively. The *Fusarium*-free region was isolated from diseased soil with polyvinyl chloride (PVC) material. Soil samples were collected from 4–6 cm soil layers in both healthy and diseased soil, denoted as ‘healthy soil’ and ‘diseased soil’, respectively. Each soil treatment had five independent experimental replicates. The soil samples were dried at room temperature, and the soil microbial function, physicochemical properties and soil enzyme were analyzed. Then, 1 Kg soil per independent experimental replicate was collected from the plot, and then divided into two subsamples for soil metagenomic sequencing and soil physicochemical property tests. Soil samples used for DNA extraction and physicochemical property tests were stored at −80 °C and 4 °C before use, respectively. (1) Determination of soil physicochemical properties: Soil organic matter (SOM) (high temperature external thermal potassium dichromate oxidation volumetric method) was tested according to NY/T1121.6-2006 [19] standard operation; pH (soil to water ratio of 2.5:1 to determine the pH value) was tested according to NY/T1121.2-2006 [20] standard operation; electrical conductivity (EC) (potential method, soil water ratio 5:1) was tested according to HJ802-2016 [21] standard operation; alkaline hydrolyzed nitrogen content (AN) (acid-base titration method) was determined according to LY/T1228-2015 [22] standard operation; available phosphorus (AP) (molybdenum-antimony resistance colorimetric method, pH ≥ 6.5) was determined according to NY/T1121.7-2014 [23] standard operation; available potassium (AK) (flame spectrophotometer) was determined according to NY/T889-2004 [24] standard operation; and available iron (AFe) and available manganese (AMn) (DTPA solution extraction method) were determined according to NY/T890-2004 [25] standard operation. Soil FDA hydrolase activity, soil urease activity, soil acid phosphatase activity and soil polyphenol oxidase activity were determined using an ELISA kit (Jiangsu Jingmei Biotechnology Co., Ltd., Yancheng, China), and the operation procedure was carried out according to the manufacturer’s instructions. (2) Soil metagenomic sequencing: The total genomic DNA was isolated from individual soil samples (0.5 g per sample) using the FastDNA Spin kit (MP Biomedical, Santa Ana, CA, USA). The total genomic DNA obtained was used to construct metagenome shotgun sequencing libraries using the Illumina TruSeq Nano DNA LT Library Preparation Kit. The libraries were sequenced using the Illumina HiSeq X-ten platform. The raw sequence reads were processed to obtain quality-filtered reads for further analyses, followed by soil microbial α diversity, principal component analysis, RDA analysis and Mantel analysis, as reported previously [18,26,27].

### 2.7. Data Processing

Microsoft Excel 2010 (14.0.4763.1150) was used to organize the data and to construct tables and graphs. All results are expressed as mean values ± standard deviation (sd). Statistical analysis was performed in SPSS 22.0. Data were evaluated by one-way analysis with Levene’s test and Student–Newman–Keuls test or Student’s *t*-test for comparisons between groups, Kruskal–Wallis for non-parametric continuous variables and three-way ANOVA analysis of variance. Probability values of less than 5% were considered to be significant.

## 3. Results

### 3.1. Isolation and Pathogenicity Identification of Fusarium Wilt Pathogen of M. charantia

**Figure 1:**
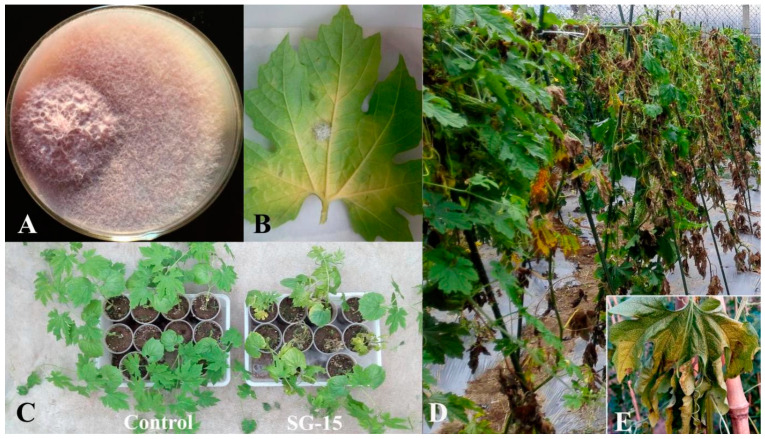
Colony morphology and pathogenicity of *F. oxysporum* strain SG−15. (**A**) Colonyl morphology of strain SG−15. (**B**) Leaf inoculated with strain SG−15 in vitro. (**C**) Pathogenicity assay in potting. (**D**) Pathogenicity assay in the field. (**E**) Typical leaf vein yellowing symptom.

In this experiment, we collected Fusarium wilt-infected *M. charantias* from growing areas in Shouguang City and Feicheng City, Shandong Province. Through tissue isolation and Koch’s postulates verification, eight pathogenic strains of *Fusarium* wilt were isolated and named SG−11, SG−12, SG−13, SG−14, SG−15, SG−16, FC−1 and FC−2. The strain SG−15 showed high virulence toward *M. charantia* (Figure 1).

**Figure 2:**
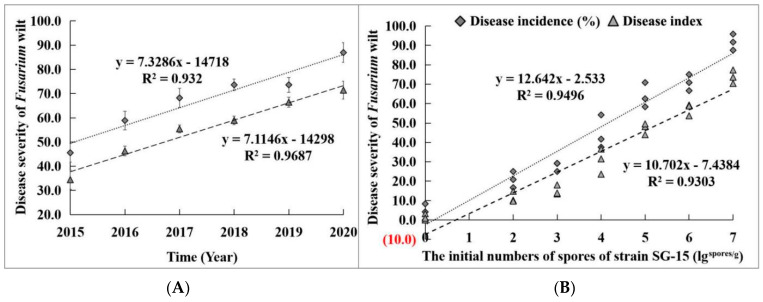
The severity of *Fusarium* wilt of *M. charantia* in the artificial disease nursery in different years (**A**) and the correlation between initial inoculum amount of strain SG−15 and the disease severity of *Fusarium* wilt (**B**).

The artificial disease nursery for *Fusarium* wilt of *M. charantia* was prepared by artificially introducing the highly virulent strain SG−15 in 2015. The incidence of *Fusarium* wilt of *M. charantia* was recorded for 45 days after transplantation. In 2015, the incidence of *Fusarium* wilt of *M. charantia* was 46.0% and the disease index was 34.0. With the extension of planting years, the occurrence of *Fusarium* wilt of *M. charantia* was increasingly severe. Moreover, by 2020, the incidence of *Fusarium* wilt of *M. charantia* in the diseased nursery reached 87.0% and the disease index reached 71.4 (Figure 2A). In addition, pot experiments showed that the incidence of *M. charantia Fusairum* wilt was linearly correlated with the initial inoculum amount of strain SG−15. When the initial inoculum concentration of strain SG−15 was 1 × 10^7^ spores·g^−1^, the incidence of *Fusarium* wilt of *M. charantia* reached 91.7% and the disease index reached 73.8 (Figure 2B).

### 3.2. Determination of Host Range of SG−15 Strain

In 2018–2020, 16 crop species including *Momordica charantia* (host plant), *Benincasa hispida*, *Cucurbita pepo*, *Luffa cylindrica*, *Cucumis melo*, *Cucumis sativus*, *Lagenaria siceraria*, *Solanum melongena*, *Lycopersicon esculentum*, *Capsicum annuum*, *Abelmoschus esculentus*, *Lablab purpureus*, *Brassica pekinensis*, *Raphanus sativus*, *Zingiber officinale* and *Ipomoea batatas* were planted in the artificial disease nursery for *Fusarium* wilt of *M. charantia*. It was found that the *Fusarium* wilt of *M. charantia* cannot infect 14 kinds of other crops except the host plant and *Lagenaria siceraria* (Table 1). Meanwhile, strain SG−15 was pathogenic to 11 *M. charantia* hybrids. However, the pathogenicity was different (Table 2). Among them, Ruyu−41 had the highest incidence (89.0%) and disease index (79.0), Ruyu−1420 had the lowest incidence (15.8%) and Ruyu−1512 had the lowest disease index (7.7).

### 3.3. Spatial Distribution of Strain SG−15 in Soil

**Figure 3:**
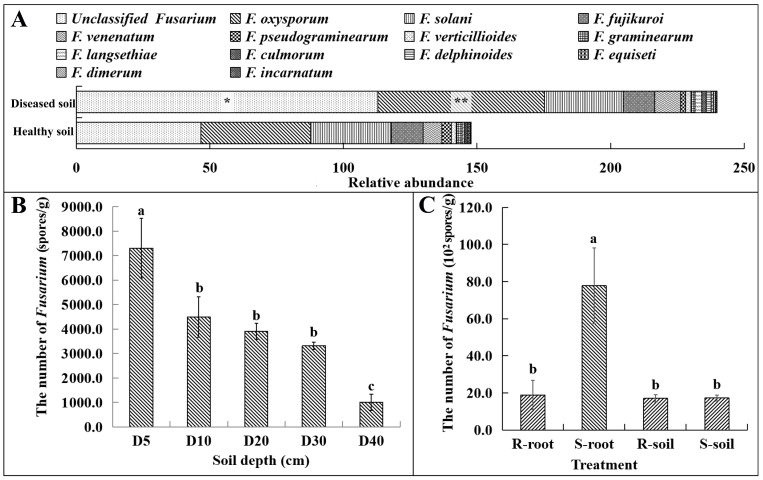
Population composition and distribution characteristics of *Fusarium* spp. in the artificial disease nursery of *Fusarium* wilt of *M. charantia*: (**A**) the abundance of *Fusarium* spp. populations in pathogenic and healthy soil based on macrogenomic data; (**B**) the number of *Fusarium* spp. in the 5–40 cm soil layer; (**C**) the number of *Fusarium* spp. in the root tissues of the host plant. R-root: the root tissue of highly disease-resistant *M. charantia*; S-root: the root tissue of highly susceptible *M. charantia*; R-soil: the rhizosphere soil of highly disease-resistant *M. charantia*; and S-soil: the rhizosphere soil of highly susceptible *M. charantia*. In the figure, * and ** indicate significant differences by *t*-test at *p* < 0.01 and *p* < 0.05 levels, respectively. Different alphabets indicate significant differences at *p* < 0.05 levels by Student–Newman–Keuls multiple comparison test.

In the soil where *M. charantia* was planted, 13 populations of *Fusarium* spp. were identified through metagenomic sequencing, with *F. oxysporum, F. solani* and *F. Fujikuroi* being the most abundant species. The population of *F. oxysporum* increased significantly in the pathogenic soil that is the soil where *Fusarium* wilt of *M. charantia* significantly increased by 51.7% in the soil where *Fusarium* wilt occurred (*p* < 0.01), compared to healthy soil. In addition, the abundance of unclassified *Fusarium* spp. in the pathogenic soil increased significantly (*p* < 0.05), by 142.4%, compared to healthy soil (Figure 3A). In the areas with a high incidence of *Fusarium* wilt, the number of *Fusarium* spp. showed a significant vertical distribution and decreased with the increase in soil depth. The *Fusarium* spp. was mainly concentrated in the 5–30 cm soil layer, and the quantity of *Fusarium* spp. in the soil layer of 5 cm was significantly higher than that in other soil layers (*p* < 0.05). There was no significant difference in the quantity of *Fusarium* spp. in the 10 cm, 20 cm and 30 cm soil layers (*p* > 0.05). In comparison, the quantity of *Fusarium* spp. in the 40 cm soil layer was significantly lower than that in the 5–30 cm soil layer (*p* < 0.05) (Figure 3B). In the artificial disease nursery for *Fusarium* wilt of *M. charantia*, the pathogen mainly existed in the root tissues of the host plant (*M. charantia*), and the number of *Fusarium* wilt pathogens was 7.8 × 10^3^ spores·g^−1^ in the highly susceptible *M. charantia* (Ruyu−41) root. The population of *Fusarium* spp. was significantly higher in the root tissues of highly susceptible *M. charantia* (Ruyu−41) than that in the root tissues (1.9 × 10^3^ spores·g^−1^) and the rhizosphere soil (1.7 × 10^3^ spores·g^−1^) of the highly resistant *M. charantia* (Ruyu−1512) (*p* < 0.05) (Figure 3C).

**Figure 4:**
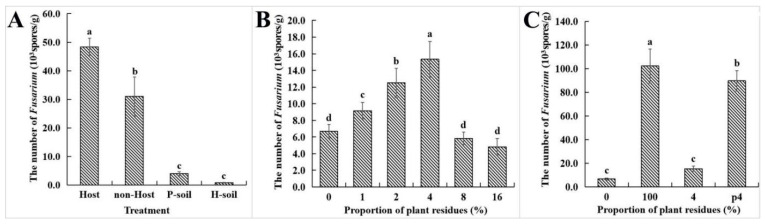
The influence of plant residues on the colonization of *F. oxysporum*. (**A**) The abundance of *F. oxysporum* in the root residue of *M. charantia* (Host), the root residue of non-host plant (Non-host), the diseased soil (P−soil) and the healthy soil (H−soil). (**B**) The influence of the adding proportion of plant residues on the abundance of *F. oxysporum* in soil. (**C**) The influence of precolonization of *F. oxysporum* in plant residues on the survival of *F. oxysporum* in soil. p4: the pathogen was added into the soil after incubation in plant debris, and the proportion of plant debris in the soil was 4%. In the figure, different letters indicate significant differences at 0.05 levels by Student–Newman–Keuls multiple comparison test.

**Figure 5:**
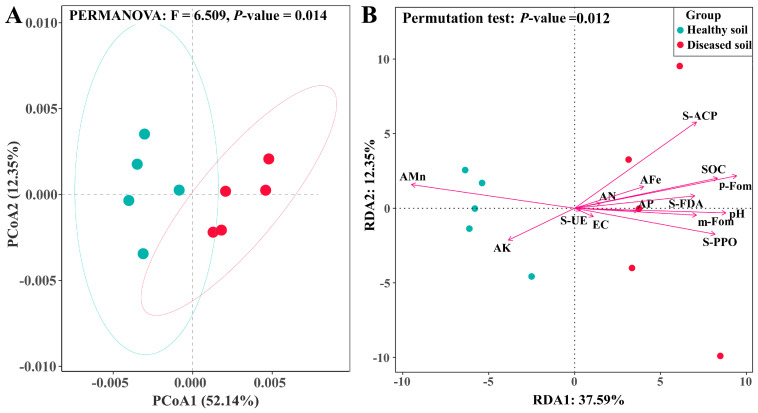
Principal component analysis of soil microbial community function (**A**) and RDA analysis. (**B**) ‘Healthy soil’, the healthy soil; ‘diseased soil’, the soil colonized by strain SG−15. m-Fom: the abundance of *F. oxysporum* populations based on macrogenomic data; p−Fom, the abundance of *F. oxysporum* populations based on the dilution plate coating method.

In the *Fusarium* wilt disease nursery for *M. charantia*, the pathogen can survive in the host plant residues. The number of *Fusarium* wilt pathogens per gram root residue of highly susceptible *M. charantia* (Ruyu−41) reached 4.8 × 10^4^ spores. The quantity of *Fusarium* spp. was significantly higher in root residues of highly susceptible *M. charantia* (Ruyu−41) compared to the rhizosphere soil (4.0 × 10^3^ spores·g^−1^) and disease-free soil (0.8 × 10^3^ spores·g^−1^) (*p* < 0.05). Meanwhile, the quantity of *Fusarium* spp. isolated from residues of non-host plant roots, such as *Benincasa hispida*, *Cucurbita pepo* and *Luffa cylindrica*, was significantly higher (3.1 × 10^4^ spores·g^−1^) compared to that in rhizosphere soil (Figure 4A). Laboratory experiments showed that the non-host plant’s debris significantly increased the colonization rate of *F. oxysporum* (Figure 4B). The soil without plant residues had 6.7 × 10^3^ spores·g^−1^ of *F. oxysporum* strain SG−15 (initial inoculation concentration was 1.0 × 10^6^ spores·g^−1^), with a colonization rate of 0.7% (Figure 5B). On the other hand, the plant residues had 1.0 × 10^5^ spores·g^−1^ of strain SG−15 with a colonization rate of 10.2% (Figure 4C). When the ratio of plant debris to soil was between 1–4%, the colonization rate of SG−15 increased significantly due to plant debris. However, when the ratio exceeded 8%, the colonization rate of SG−15 was inhibited. This could be the presence of other microorganisms in the soil that inhibited SG−15 colonization of (Figure 4B). Also, when the pathogen was added to the soil after colonizing diseased plant residues, its colonization rate in the soil was significantly increased. The colonization rate of SG−15 in the p4 treatment was 5.8 times higher than that in the control treatment (the pathogen and plant residues were added to the soil at the same time).

### 3.4. Effects of Colonization of Strain SG−15 on Soil Physicochemical Properties

**Figure 6:**
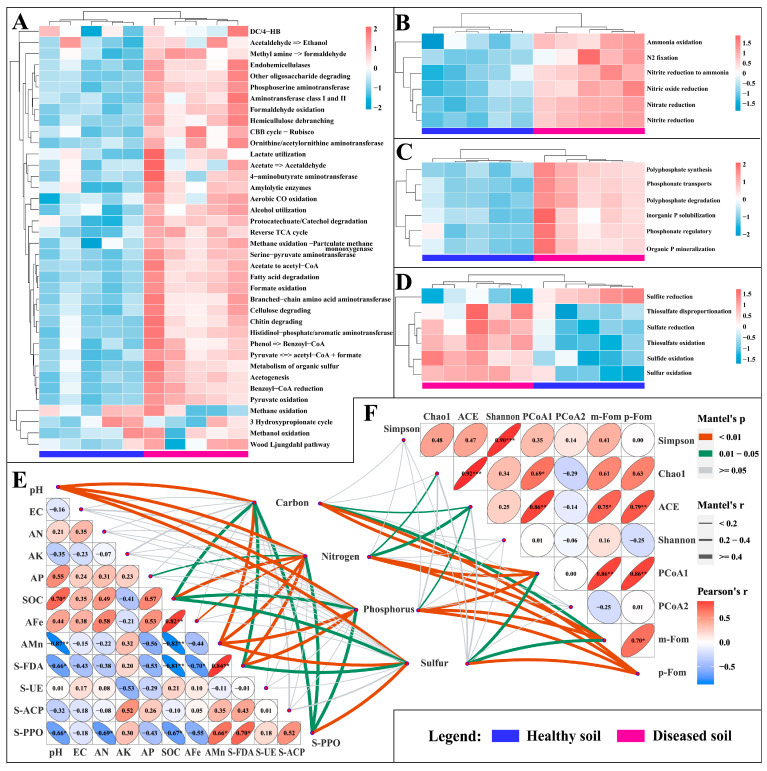
Variation in the cycling of C, N, P and S based on soil microbial activity mediated by strain SG−15. The heatmap characterizes the relative abundance of metabolic pathways in sections A, B, C and D: red represents high relative abundance, while blue represents low relative abundance. (**A**) Soil carbon cycling. (**B**) Soil nitrogen cycling. (**C**) Soil phosphorus cycling. (**D**) Soil sulfur cycling. (**E**) Mantel analyzed the correlation between soil physical and chemical properties and C, N, P and S cycles. (**F**) Mantel analyzed the correlation between soil microbial diversity index and the relative abundance of *Fusarium oxysporum* and C, N, P and S cycles. The symbol * indicates significant correlation at *p* < 0.05 level by Spearman-related analysis, ** indicate significant correlation at *p* < 0.01 level by Spearman-related analysis and *** indicate significant correlation at *p* < 0.001 level by Spearman-related analysis.

The physicochemical properties and soil enzyme activities of the soil uncolonized by strain SG−15 (healthy soil) and the soil colonized by strain SG−15 (diseased soil) differed (Table 3). Compared with the ‘healthy soil’ treatment, organic matter content (SOC), pH, electrical conductivity (EC), available nitrogen content (AN), available phosphorus content (AP), available iron content (AFe), acid phosphatase activity (ACP), fluorescein diacetate activity (FDA), polyphenol oxidase activity (PPO) and urease activity (UE) in the ‘diseased soil’ treatment increased, while the content of available potassium (AK) and available manganese (AMn) decreased in soil colonized by S-Fom. Among them, SOC (57.70%), pH (1.95%), AFe (27.71%), ACP (30.51%), FDA (42.46%) and PPO (23.00%) were significantly increased in the soil, whereas AMn content was decreased by 37.80% (*p* < 0.05). Results of the Spearman correlation analysis showed a significant negative correlation between soil AMn content and the relative abundance of *F. oxysporum* (*p* < 0.05). In contrast, the relative abundance of *F. oxysporum* was significantly positively correlated with soil pH, AFe, SOC, FDA, and PPO (*p* < 0.05). Based on the Pearson correlation analysis, soil FDA activity was positively correlated with soil pH while negatively correlated with AMn content. Also, PPO activity was positively correlated with soil pH and SOC content but negatively correlated with AMn content (Figure 6E).

### 3.5. Effects of Colonization of Strain SG−15 on Soil Microbial Function

Analysis of microbial community structure revealed that the presence of *F. oxysporum* strain SG−15 influenced soil microbial community function. Permanova analysis results showed that the microbial community function (F = 6.509, *p* = 0.014) in soil uncolonized by strain SG−15 (healthy soil) and soil colonized by strain SG−15 (diseased soil) could be significantly distinguished in a non-binding principal coordinate analysis (PCoA) (Figure 5A). Based on Bray–Curtis distance, the first axis of PCoA (PCoA1) explained 52.14% of the community functional variation, and the second axis (PCoA2) explained 21.20% of the community functional variation. ‘Healthy soil’ and ‘diseased soil’ had a total of 9758 functional groups. Compared with ‘healthy soil’, 568 functional groups were degraded and 357 functional groups were added in ‘diseased soil’. Redundancy analysis (RDA) results showed that soil pH value, organic matter content (SOC), available manganese content (AMn), FDA hydrolase activity (FDA), acid phosphatase activity (ACP), polyphenol oxidase activity (PPO) and *Fusarium oxysporum* abundance had significant effects on the function and distribution of the microbial community (Figure 5B and Table 4). They may be an essential environmental factor in the transition from the function of a healthy soil microbial community to that of a pathogenic soil microbial community. It was further verified that the relative abundance of *F. oxysporum* was positively correlated with soil pH value, organic matter content (SOC), available nitrogen content (AN), available phosphorus content (AP), available iron content (AFe), the FDA activity, PPO activity, ACP activity and negatively correlated with available manganese content (AMn) and available potassium content (AK) (Figure 5B).

### 3.6. Effects of Colonization of Strain SG−15 on Microbe-Mediated C, N, P and S Cycles in Soil

The colonization of strain SG−15 altered the soil’s C, N, P and S cycling pathways, the relative abundance of genes and enhanced the metabolic function (Figure 6A–D). The soil carbon cycling involved organic carbon oxidation (cellulose degrading, hemicellulose debranching, amylolytic enzymes, endohemicellulases, other oligosaccharide degrading, protocatechuate/catechol degradation, fatty acid degradation, chitin degrading, etc.), carbon fixation (Wood–Ljungdahl pathway, reverse TCA cycle, DC/4-HB, CBB cycle rubisco, etc.), fermentation (pyruvate<=>acetyl-CoA+formate, acetate to acetyl-CoA, acetogenesis, lactate utilization, pyruvate oxidation, alcohol utilization, etc.) and methane oxidation. The soil nitrogen cycling mainly involved nitrogen fixation, nitrite ammonification, ammonia oxidation, nitrate reduction, nitric oxide reduction and nitrite reduction (nitrite reduction to ammonia). Soil phosphorus cycling involved organic phosphorus mineralization, inorganic phosphorus solubilization, polyphosphate degradation, polyphosphate synthesis, phosphate transports and phosphate regulation. Soil sulfur cycling is mainly involved in dissimilatory sulfur metabolism (thiosulfate disproportionation, sulfite reduction, thiosulfate oxidation, sulfide oxidation, sulfate reduction, etc.) and sulfur oxidation. Results of the Mantel test showed significantly positive correlations between soil C, N, P and S cycles and soil pH, SOC, AMn, FDA, PPO, soil microbial ACE diversity index, soil microbial beta diversity index (PCoA1) and the abundance of *Fusarium oxysporum* (Figure 6E,F).

## 4. Discussion

### 4.1. Isolation and Pathogenicity Determination of Fusarium wilt of M. charantia

In recent years, the development of protected vegetable cultivation in China has increased soil-borne diseases such as *Fusarium* wilt, *Verticillium* wilt, root rot disease and root-knot nematodes disease. These diseases have become more severe and are now critical factors that restrict the development of facility vegetables due to the fixity and limitation of intensive vegetable production and cultivation, creating a stable internal micro-ecological environment suitable for soil-borne diseases. *Fusarium* wilt of gourd vegetables caused by pathogenic *F. oxysporum* is widespread in 18 provinces and cities, including Jilin, Liaoning, Shandong, Hubei, and Beijing city, with annual losses of about 10%~30%, or even complete loss has been reported. From 2014 to 2015, we investigated bitter gourd’s main growing areas in Feicheng and Shouguang City, Shandong Province. We found a large-scale outbreak of *Fusarium* wilt of *M. charantia* in our province, resulting in a loss rate of over 30% for greenhouses with severe *Fusarium* wilt. Based on Koch’s postulates, we isolated and obtained seven strains from *M. charantia* in Shouguang City and Feicheng City, Shandong Province, identifying them as the pathogens of the *Fusarium* wilt of *M. charantia*. Based on the genetic distance and genetic similarity coefficient among the population of *F. oxysporum* f. sp. *momordicae*, we found that strain SG−15 and the other seven strains were most closely related to the pathogen causing *Fusarium* wilt of *M. charantia* [27].

### 4.2. Mechanism of Fusarium wilt Outbreaks in Continuous Cropping Soil

*Fusarium* wilt caused by pathogenic *Fusarium oxysporum* is a highly destructive plant disease that is increasingly causing global losses year after year [1,28]. The occurrence of crop *Fusarium* wilt was significantly correlated with the number of pathogens in rhizosphere soil. When the concentration of pathogens in soil was lower than 10^3^ spores·g^−1^, the symptoms of *Fusarium* wilt were not obvious. When the population of *F. oxysporum* in soil reached 10^3^~10^5^ spores·g^−1^, the severity of *Fusarium* wilt increased [29]. The accumulation and long-term existence of pathogenic *F. oxysporum* in soil directly induced *Fusarium* wilt. The accumulation and long-term existence of the *Fusarium* wilt pathogen in soil may be due to a lack of disease-resistant crop varieties and intensified agricultural production.

Lack of disease-resistant varieties. At present, breeding and utilization of disease-resistant varieties are the most effective and economic measures to control plant diseases [30]. Extensive and persistent resistance is an important goal of resistance breeding, but the lack of effective disease-resistant varieties, especially extensive and persistent disease-resistant varieties, limits the development of persistent resistance breeding. Now, wild and semi-cultivated *M. charantia* species in China show high resistance to *Fusarium* wilt, but a lack of highly *Fusarium* wilt-resistant *M. charantia* materials, which may only account for 0.7% of the resources of *M. charantia* [31,32,33]. Our study showed that the quantity of *Fusarium* spp. in the root tissue of susceptible *M. charantia* was significantly higher than that in the root tissue of disease-resistant varieties. This indicated that a large number of susceptible varieties were planted to provide a breeding ground for the *Fusarium* wilt pathogen to proliferate in the soil. Meanwhile, the *Fusarium* wilt pathogen can spread through the soil using the roots of infected plants. When the host plant dies, *F. oxysporum* releases spores into the soil after the roots infected with it decay. The lack of *M. charantia* with high resistance or immunity to *Fusarium* wilt limited the breeding of cultivars with high resistance to *Fusarium* wilt, which provided convenient conditions for the growth and propagation of the *Fusarium* wilt pathogen in soil and aggravated the occurrence of *Fusarium* wilt. Therefore, it is still necessary to strengthen the collection, introduction, innovation, identification, and evaluation of resistance resources of *Fusarium* wilt of *M. charantia* and the study on the mechanism of resistance to *Fusarium* wilt.

Intensive production mode. *Fusarium oxysporum* exists widely in native soil worldwide and can be found in plant roots and abiotic organic matter [8,34]. *Fusarium oxysporum* generally does not cause disease in native plants, and crop cultivation does not significantly alter native populations of *F. oxysporum*. However, pathogenic strains of *F. oxysporum* can originate from these native populations, and continuous cropping provides more opportunities for pathogen evolution [8]. As a filamentous fungus with very strong saprophytic ability, *F. oxysporum* is nearly impossible to eliminate once it establishes in the soil [35].

Soil microflora, including microbial biomass, community, diversity and the abundance of specific microbiota, is considered critical for maintaining soil health, soil quality, soil enzyme activity and disease suppression [36]. High soil microbial biodiversity and dynamic balance of microbiota are the important basis for inhibiting pathogen invasion. Long-term continuous monocropping can lead to the accumulation of pathogenic microorganisms in soil, which will become the dominant population in soil, and eventually lead to the imbalance of soil microbiota. The soil colonization process of pathogens such as the *Fusarium* wilt pathogen (*F. oxysporum*) causes drastic changes in the native soil microbial community, which are manifested as the increase in the number of plant pathogenic microorganisms and changes in microbial diversity and community function [15]. The prevalence of *Fusarium* wilt was significantly positively correlated with the abundance and diversity of soil fungi. Intensive monoculture resulted in an increase in the abundance and diversity of soil fungi and a decrease in the abundance and diversity of soil bacteria, as well as a decrease in the ability of soil microflora to resist the invasion of *Fusarium* spp., ultimately leading to the successful colonization and accumulation of *Fusarium* spp. in the soil [37]. The abundance of *Fusarium* spp. was higher in the soil with high *Fusarium* wilt incidence than that in non-pathogenic soil, and the abundance and diversity of fungi or bacteria may be related to the abundance of *Fusarium* spp. [15].

### 4.3. Effects of the Fusarium Wilt Pathogen Colonization on Soil Chemical Properties

Changes in soil chemical properties can promote the growth and propagation of the *Fusarium* wilt pathogen, which increases the disease severity [35,38]. Soil chemical properties like nitrogen (N), phosphorus (P), potassium (K), calcium (Ca), magnesium (Mg), zinc (Zn), iron (Fe) and manganese (Mn) can all affect the severity of *Fusarium* wilt [35]. The available iron (AFe) content in soil is high in areas with a high incidence of banana *Fusarium* wilt [14]. The available phosphorus (AP), calcium (Ca), magnesium (Mg) and pH in healthy soil were significantly higher than those in pathogenic soil. The higher pH and available phosphorus (AP) content inhibited the occurrence of *Fusarium* wilt [15]. Although soil chemical properties can significantly affect the occurrence of *Fusarium* wilt, it is not sufficient to inhibit the growth and propagation of pathogenic microorganisms in the soil. There may be a feedback loop between soil properties and microbial communities during the transformation process from *Fusarium* wilt disease-suppressing soil to disease-conductive soil [39]. Soil chemical properties promoted the change of soil microbial community, and the changed microbial community in turn may contribute to the improvement of soil’s resistance to *Fusarium* wilt; that is, soil chemical properties reduced the incidence of *Fusarium* wilt by improving microbial activity. However, our experimental results demonstrated that the accumulation of *Fusarium* wilt pathogens can also affect the normal soil elements cycling (such as carbon, nitrogen, phosphorus, sulfur, etc.), and thus affect the soil’s chemical properties.

Soil is a complex mix; plant residues are the main source of soil nutrients and the main activity site for soil microorganisms. “Soil microorganism organic matter” is essential in maintaining soil function and health through a combination of soil nutrient cycling and energy flow. The quantity and quality of input of plant organic matter affect not only the physical and chemical properties of soil, but also the microbial biomass, diversity, community structure and biochemical activities [36]. Microorganisms and organic matter interact in the soil. The composition and activity of soil microbes affect the decomposition of organic matter. In contrast, the dynamic change of soluble organic material, in turn, affects the composition of the microbial community [40]. Crop disease residues in the soil increase the *Fusarium* wilt pathogen population and may accelerate plant residue decomposition [11]. We speculated that the pathogen causing *Fusarium* wilt may affect the soil nutrient cycling by mediating the decomposition process of plant residues, particularly diseased ones, thereby altering soil chemical properties.

## 5. Conclusions

The pathogen causing the *Fusarium* wilt of *M. charantia* in the main growing area of Shandong Province was the *F. oxysporum* f. sp. *momordicae*, and strain SG−15 was a highly virulent strain with host specialization. It was found that the *Fusarium* wilt pathogen was distributed in 0–40 cm soil, mainly in crop residues in 0–10 cm soil by preparing an artificial disease nursery for *Fusarium* wilt of *M. charantia*. At the same time, the susceptible host plants were the primary medium for the *Fusarium* wilt pathogen to grow and reproduce in soil. In contrast, the disease-resistant host plants and non-host crops were not conducive to the growth and reproduction of the *Fusarium* wilt pathogen. After harvest, the *Fusarium* wilt pathogen could survive in residues of susceptible host plants, disease-resistant host plants and non-host crops. The *Fusarium* wilt pathogen also affects soil nutrient cycling by mediating the decomposition process of plant residues (especially diseased plant residues). It thus leads to a change in soil chemical properties.

## Figures and Tables

**Table 1:** The pathogenicity of strain SG−15 on host plant and non-host plants.

Crops	Cultivated Varieties	Number	Incidence (%)	Disease Index
*Momordica charantia*	Ruyu−41	51	100	81.82
*Benincasa hispida*	Heihu	51	0.00	0
*Cucurbita pepo*	Cuibao F1	51	0.00	0
*Luffa cylindrica*	/	51	0.00	0
*Cucumis melo*	Lvbaoshi	51	0.00	0
*Cucumis sativus*	Yousheng	51	0.00	0
*Lagenaria siceraria*	/	51	75.76	59.60
*Solanum melongena*	/	51	0.00	0
*Lycopersicon esculentum*	Weinisi F1	51	0.00	0
*Capsicum annuum*	Lashen F1	51	0.00	0
*Abelmoschus esculentus*	Shouhe	51	0.00	0
*Lablab purpureus*	Baimeng	51	0.00	0
*Brassica pekinensis*	Jinqing 1	51	0.00	0
*Raphanus sativus*	/	51	0.00	0
*Zingiber officinale*	Lujiang 1	51	0.00	0
*Ipomoea batatas*	Yanshu 25	51	0.00	0

**Table 2:** The pathogenicity of strain SG−15 on different cultivated varieties of *M. charantia*.

Cultivated Varieties	Incidence (%)	Disease Index	Relative Disease Resistance (%)
Ruyu−1420	15.79 ± 5.26 e	11.50 ± 2.88 ef	85.49 ± 3.16
Ruyu−1510	42.31 ± 6.66 c	34.90 ± 4.19 c	55.45 ± 7.36
Ruyu−1511	15.34 ± 7.33 e	10.76 ± 5.80 ef	86.73 ± 5.80
Ruyu−1512	21.79 ± 4.75 de	7.66 ± 1.39 f	90.26 ± 1.86
Ruyu−1515	57.73 ± 1.05 b	51.42 ± 3.95 b	34.54 ± 7.24
Ruyu−1516	32.03 ± 2.26 cd	26.60 ± 1.57 d	66.21 ± 1.65
Ruyu−1522	63.48 ± 3.01 b	53.58 ± 6.76 b	31.84 ± 9.86
Ruyu−1523	31.23 ± 3.90 cd	21.83 ± 5.34 de	71.81 ± 9.23
Ruyu−1534	25.40 ± 2.75 de	21.87 ± 3.36 de	72.19 ± 4.65
Ruyu−1536	33.77 ± 4.50 cd	18.05 ± 3.72 def	77.06 ± 4.91
Ruyu−41	89.02 ± 8.08 a	78.95 ± 7.62 a	/

In the table, different letters indicate significant differences at 0.05 levels by Student–Newman–Keuls multiple comparison test.

**Table 3:** Effect of strain SG−15 invasion on soil chemical properties, enzyme activity and soil microbial α diversity.

Treatment		Healthy Soil	Diseased Soil	Spearman Correlation Analysis	
				m-Fom	p-Fom
Soil chemical properties	pH	6.68 ± 0.07	6.81 ± 0.06 *	0.778 **	0.607
	EC (ms/m)	4.40 ± 0.70	4.80 ± 0.22	0.539	0.474
	AN (mg/kg)	69.96 ± 12.25	78.80 ± 9.06	0.395	0.14
	AK (mg/kg)	67.92 ± 11.03	56.80 ± 9.83	−0.091	−0.456
	AP (mg/kg)	31.32 ± 3.68	33.94 ± 1.00	0.539	−0.043
	SOC (g/kg)	14.42 ± 1.82	22.74 ± 2.04 **	0.818 **	0.626
	AFe (mg/kg)	27.43 ± 1.05	35.03 ± 6.05 *	0.818 **	0.663 *
	AMn (mg/kg)	9.18 ± 1.14 **	5.71 ± 0.88	−0.830 **	−0.736 *
Soil enzyme activity	S-FDA (μmol/g/d)	190.46 ± 20.96	248.56 ± 50.25 *	0.685 *	0.699 *
	S-ACP (μmol/g/d)	8.84 ± 1.36	12.59 ± 2.88 *	0.648 *	0.541
	S-PPO (mg/g/d)	950.4 ± 74.06	1169.02 ± 81.17 **	0.839 **	0.652 *
	S-UE (μmol/g/d)	1636.94 ± 149.84	1677.01 ± 278.18	0.345	0.073
Soil microbial α diversity	Simpson	0.987 ± 0.001	0.987 ± 0.001	/	/
	Chao1	8894.9 ± 111.7	9143.64 ± 154.48 *	/	/
	ACE	8911.33 ± 81.54	9167.01 ± 102.08 *	/	/
	Shannon	8.47 ± 0.04	8.47 ± 0.04	/	/
Relative abundance of *F. oxysporum*	m-Fom	38.8 ± 4.22	62.23 ± 9.85 **	/	/
	p-Fom	4400 ± 2073.64	20200 ± 3633.18 **	/	/

Diseased soil, the soil colonized by strain SG−15. m-Fom: the abundance of *F. oxysporum* populations based on macrogenomic data; p-Fom: the abundance of *F. oxysporum* populations based on the dilution plate coating method. The symbol * indicates significant difference or significant correlation at *p* < 0.05 level by independent T-test or Spearman-related analysis and ** indicate significant difference or significant correlation at *p* < 0.01 level by independent T-test or Spearman-related analysis.

**Table 4:** Determination of the functional variation of soil microbial communities based on biotic and abiotic environmental variables based on a redundancy analysis model with unrestricted permutation tests.

Environmental Factors	RDA1	RDA2	R2	*p* Value
pH	0.9994	−0.0356	0.7693	0.0080 **
EC	0.8865	−0.4628	0.1072	0.6782
AN	0.9763	0.2163	0.1335	0.6167
AK	−0.8744	−0.4851	0.3856	0.1999
AP	0.9990	−0.0458	0.3239	0.2574
SOC	0.9718	0.2359	0.7476	0.0080 **
AFe	0.9408	0.3389	0.3738	0.2094
AMn	−0.9864	0.1645	0.8410	0.0020 **
S-FDA	0.9931	0.1177	0.6151	0.0320 *
S-ACP	0.7742	0.6330	0.7867	0.0050 **
S-PPO	0.9780	−0.2085	0.7300	0.0105 *
S-UE	0.8948	−0.4465	0.0327	0.8801
m-Fom	0.9978	−0.0657	0.6211	0.0335 *
p-Fom	0.9745	0.2245	0.8458	0.0040 **

The symbol * indicates significant influence at *p* < 0.05 level by permutation test and ** indicate significant influence at *p* < 0.01 level bypermutation test.

## Data Availability

Data will be made available on request. The raw Illumine sequence data of metagenomic data have been deposited in the sequence read archive (SRA submission: SUB9063041) at NCBI under Bioproject accession #PRJNA701786. The data that support the findings of this study are available from the corresponding author upon reasonable request.
